# Increase in the astaxanthin synthase gene (*crtS*) dose by *in vivo* DNA fragment assembly in *Xanthophyllomyces dendrorhous*

**DOI:** 10.1186/1472-6750-13-84

**Published:** 2013-10-09

**Authors:** Gabriela Contreras, Salvador Barahona, María Cecilia Rojas, Marcelo Baeza, Víctor Cifuentes, Jennifer Alcaíno

**Affiliations:** 1 Departamento de Ciencias Ecológicas, Facultad de Ciencias, Universidad de Chile, Las Palmeras 3425, Casilla, Santiago 653, Chile; 2 Departamento de Química, Facultad de Ciencias, Universidad de Chile, Las Palmeras 3425, Casilla, Santiago 653, Chile

**Keywords:** *Xanthophyllomyces dendrorhous*, Astaxanthin synthase, DNA assembler

## Abstract

**Background:**

*Xanthophyllomyces dendrorhous* is a basidiomycetous yeast that is relevant to biotechnology, as it can synthesize the carotenoid astaxanthin. However, the astaxanthin levels produced by wild-type strains are low. Although different approaches for promoting increased astaxanthin production have been attempted, no commercially competitive results have been obtained thus far. A promising alternative to facilitate the production of carotenoids in this yeast involves the use of genetic modification. However, a major limitation is the few available molecular tools to manipulate *X. dendrorhous*.

**Results:**

In this work, the DNA assembler methodology that was previously described in *Saccharomyces cerevisiae* was successfully applied to assemble DNA fragments *in vivo* and integrate these fragments into the genome of *X. dendrorhous* by homologous recombination in only one transformation event. Using this method, the gene encoding astaxanthin synthase (*crtS*) was overexpressed in *X. dendrorhous* and a higher level of astaxanthin was produced.

**Conclusions:**

This methodology could be used to easily and rapidly overexpress individual genes or combinations of genes simultaneously in *X. dendrorhous*, eliminating numerous steps involved in conventional cloning methods.

## Background

*Xanthophyllomyces dendrorhous* is a basidiomycetous yeast with potential in the biotechnology industry as it is able to synthesize carotenoids, particularly astaxanthin. Carotenoids are natural yellow, orange, or red pigments, and more than 700 different carotenoid chemical structures have been described to date [1]. Animals are unable to synthesize these pigments *de novo* and can only obtain them in their diet. These pigments are currently used as food colorants and have received attention for their ability to alleviate chronic diseases due to their antioxidant properties, which can mitigate the damaging effects of oxidative stress induced by reactive oxygen species (ROS) [2]. Among carotenoids, astaxanthin (3,3′-dihydroxy-β,β-carotene-4-4′-dione) is notable based on its antioxidant properties, which are greater than those of beta-carotene or even alpha-tocopherol [3], and the application of astaxanthin in the pharmaceutical and cosmetic industries has recently been explored [4]. Astaxanthin has also been widely used in the aquaculture industry as a colorant for cultured salmonids to achieve the flesh color that is preferred by consumers. In addition, astaxanthin is an essential nutritional component for proper fish growth and reproduction, making this compound a significant factor in aquaculture production costs [4].

**Figure 1:**
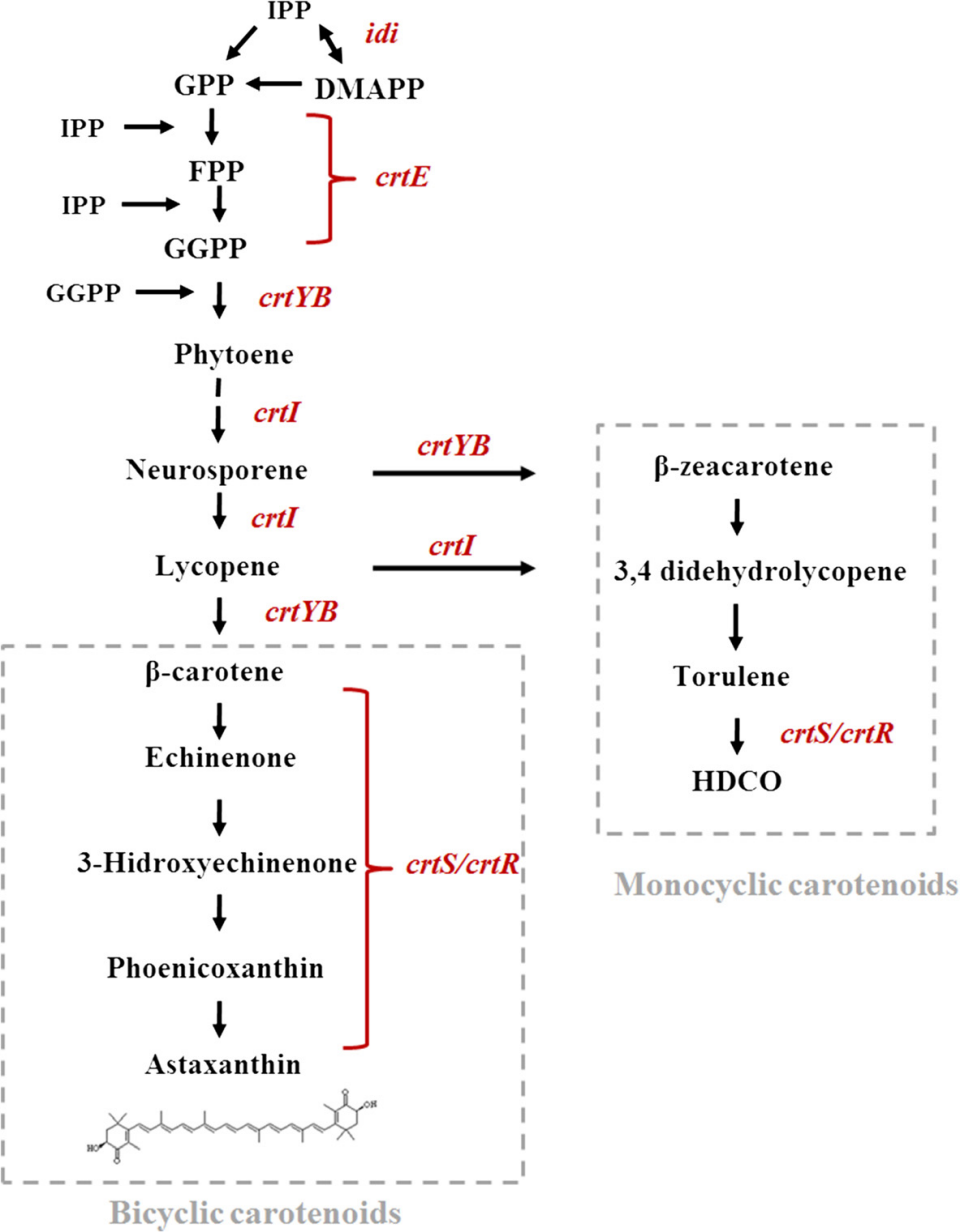
**Diagram of the astaxanthin biosynthetic pathway in *X. dendrorhous*.** The astaxanthin biosynthesis pathway in *X. dendrorhous* proposed by [21] and [22]. Metabolite abbreviations: IPP (isopentenyl pyrophosphate), DMAPP (dimethylallyl pyrophosphate), GPP (geranyl pyrophosphate), FPP (farnesyl pyrophosphate), GGPP (geranylgeranyl pyrophosphate) and HDCO (3-hydroxy-3′,4′-didehydro-β,ψ-carotene-4-one). The names of the genes controlling each step are written in red: *idi* (IPP-isomerase), *crtE* (geranylgeranyl pyrophosphate synthase), *crtYB* (PBS, phytoene-β-carotene synthase), *crtI* (PDS, phytoene desaturase), *crtS* (astaxanthin synthase) and *crtR* (cytochrome P450 reductase).

*X. dendrorhous* is one of the few microorganisms that produces astaxanthin. However, the production of this pigment by wild-type strains is too low (200–400 μg per g of dry yeast) to provide a natural source that is economically competitive with chemical synthesis of this pigment. Therefore, many efforts have attempted to improve the astaxanthin production from *X. dendrorhous*, including optimization of culture conditions such as glucose concentration [5,6], oxygen levels [6,7], pH [8,9], carbon/nitrogen ratio [10], and light intensities [11], in addition to classic random mutagenesis methods [12-15]. A promising alternative to increase the astaxanthin yield in this yeast is to overexpress the genes involved in carotenoid synthesis (Figure 1) for which several attempts have been performed (For a review see: [16]). A small increase in the amount of total carotenoids was obtained by overexpressing the gene encoding phytoene synthase-lycopene cyclase (*crtYB*) by integrating multiple copies of this gene into the ribosomal DNA [17]. However, this increase was mainly due to increased synthesis of beta-carotene and echinenone, while the astaxanthin content was slightly reduced (or unaffected). In another experiment in which the gene encoding phytoene desaturase (*crtI*) was overexpressed, the overall carotenoid production decreased including a 50% reduction in the astaxanthin fraction [17]. Similarly, the overexpression of the gene encoding isopentenyl pyrophosphate isomerase (*idi*) led to a decrease in the amount of total carotenoids [18]. In another work attempting to promote metabolite flow towards carotenoid biosynthesis, the cDNA encoding the geranylgeranyl pyrophosphate synthase gene (*crtE*) was overexpressed, resulting in a strain with slightly higher carotenoid levels, although the astaxanthin levels were not increased [19]. However, the simultaneous insertion of extra copies of the *crtYB* and *crtS* (which encodes the astaxanthin synthase) genes in an astaxanthin-overproducing strain obtained by random mutagenesis resulted in transformants with an even higher astaxanthin content [20]. Therefore, the overexpression of a single gene is not enough to significantly increase the astaxanthin level in *X. dendrorhous,* and overexpression of different combinations of carotenogenic genes seemed to provide better results.

One of the major limitations in the genetic manipulation of *X. dendrorhous* is the limited number of molecular tools available to transform and engineer this yeast. Recently, an *in vivo* method for DNA fragment assembly and yeast transformation, DNA assembler, was reported [23]. With this technique, numerous DNA fragments are assembled *in vivo* via homologous recombination at their ends following a single transformation event, allowing the incorporation of an entire heterologous biochemical pathway in *Saccharomyces cerevisiae*. As the *X. dendrorhous* homologous recombination machinery has previously been successfully exploited for the development of transformation strategies and gene function analysis [17,24-27], in this work, we adapted the DNA assembler methodology to overexpress the gene encoding astaxanthin synthase (*crtS*) that catalyzes the formation of astaxanthin from beta-carotene [28,29] in *X. dendrorhous*.

## Results

### *In vivo* assembly and integration of DNA fragments into the genome of *X. dendrorhous*

To evaluate the feasibility of assembling DNA fragments *in vivo* in *X. dendrorhous*, a DNA cassette containing only the gene that confers resistance to hygromycin B [30] was integrated in the genome of the wild-type *X. dendrorhous* strain UCD 67–385. A 1,260 bp locus called *DHS3* [GenBank: JN835289.1] was chosen as one of the potential target site, which is located at 2,110 bp downstream of the *X. dendrorhous HIS3* gene. This region is transcribed and encodes an uncharacterized gene product, so we expected that its interruption would not drastically affect the physiology of the yeast.

**Figure 2:**
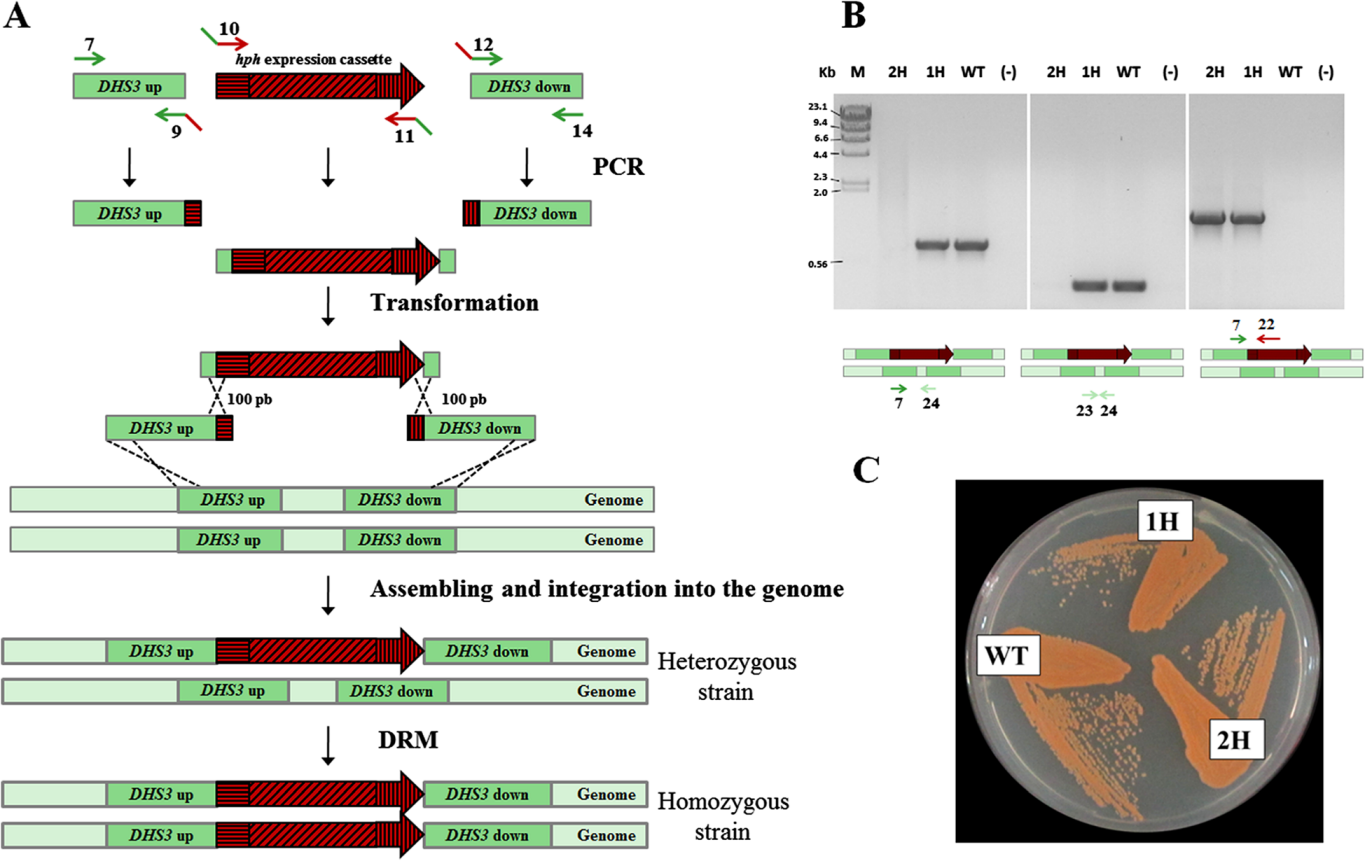
**Assembly and integration of the hygromycin B resistance cassette into the genome of *X. dendrorhous *by DNA assembler. A)** Three DNA fragments, *DHS3* “up”, *hph* expression cassette (composed by EF-1α promoter, *hph* gene and *gpd* terminator) and *DHS3* “down” were amplified by PCR to direct the integration of the hygromycin B cassette into the *DHS3* locus of the *X. dendrorhous* genome. Then, they were co-transformed, assembled (by recombination at their overlapping ends) and integrated into the *X. dendrorhous* genome. The arrows represent the primers used and the black crosses represent the *in vivo* homologous recombination event. By this way, the heterozygous strain was obtained, which was submitted to the double recombinant method (DRM) to obtain the homozygous strain. **B)** Evaluation of the hygromycin B cassette integration by PCR of strains *Xd_*1H (1H, one hygromycin B cassette copy), *Xd_*2H (2H, two hygromycin B cassette copies) and parental UCD 67–385 (WT), and control without DNA (-). A scheme representing the primer sets (in arrows and numbers according to Additional file 1: Table S1) that were used and the DNA target are included under each gel photograph. M: Molecular marker, lambda DNA digested with *Hind*III. **C)** Color phenotype of strains *Xd_*1H (1H), *Xd_*2H (2H) and parental UCD 67–385 (WT) grown on an MM_V_ agar plate.

Three DNA fragments (454 bp *DHS3* “up” for upstream, 1,817 bp hygromycin B resistance cassette and 460 bp *DHS3* “down” for downstream) were prepared as illustrated in Figure 2A. First, each fragment was individually PCR amplified with a set of primers designed to make the 5′ end of the fragment overlap the 3′ end of the preceding DNA fragment. In this way, the hygromycin B resistance cassette overlaps the two flanking DNA fragments targeting the *DHS3* locus. The overlapping region contained approximately 100 bp of sequence homology between fragments to allow *in vivo* homologous recombination between them. A diploid *X. dendrorhous* wild-type strain [31] was co-transformed with the three DNA fragments by electroporation. Assembly of these fragments and their integration into the genome was accomplished, as nine hygromycin B-resistant transformants were obtained. PCR analyses confirmed that all of them contained the resistance cassette at the expected integration target. However, as the starting strain UCD 67–385 is diploid [31], a wild-type *DHS3* allele was still detected in the resulting transformants, indicating that they were heterozygous at this locus. For this reason, one of the transformants was randomly chosen (named *Xd_*1H, for one hygromycin-resistance cassette copy) to obtain the homozygous strain *Xd_*2H (2H for two copies of the hygromycin-resistance cassette) using the double recombinant method (DRM) [30] (Figure 2A). Panel B of Figure 2 shows the amplicons obtained from genomic PCR confirming the integration of the hygromycin B resistance cassette into the expected locus in strains *Xd_*1H (*DHS3*/*dhs3*^::*hph*^) and *Xd_*2H (*dhs3*^::*hph*^/*dhs3*^::*hph*^). To the naked eye, the color of the heterozygous and homozygous strains is identical to the wild-type strain and both strains are able to grow in minimal medium (Figure 2C). Total carotenoids were quantified and no significant differences were detected between the transformants and the wild-type strain (data not shown). Thus, the interruption of the *DHS3* locus did not cause auxotrophy or greatly affected the carotenogenesis in this yeast.

To confirm that this transformation methodology is effective for other integration targets, the resistance cassette was successfully integrated into two other genomic loci. However, the color phenotype of the transformants was different from the parental strain (data not shown).

### Increase of *crtS* gene dose

**Figure 3:**
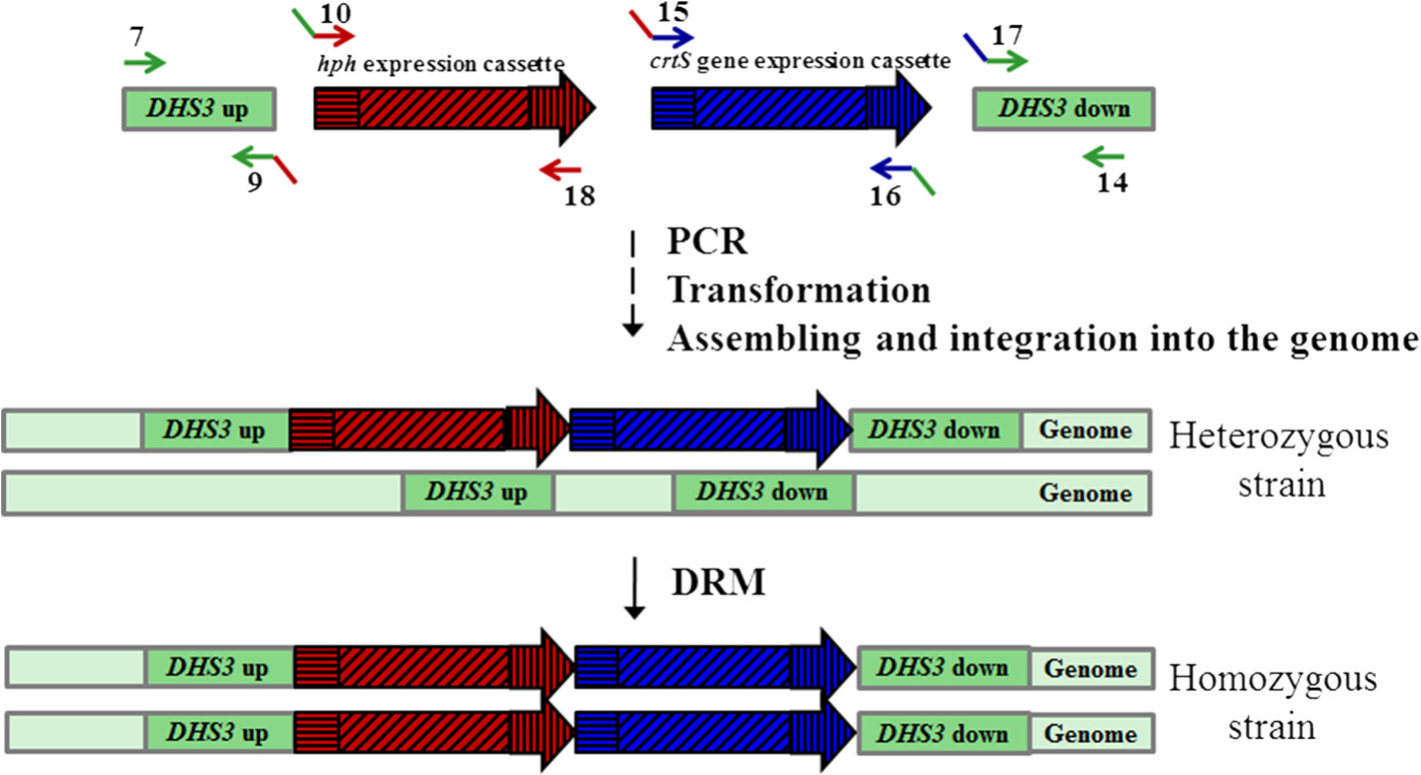
**Assembly and integration of hygromycin B resistance and *crtS *gene expression cassettes into the *DHS3 *locus of *X. dendrorhous *by DNA assembler.** Four DNA fragments: *DHS3* “up”, *hph* expression cassette (composed by EF-1α promoter, *hph* gene and *gpd* terminator), *crtS* gene expression cassette (composed by EF-1α promoter, *crtS* gene and *actin* transcription terminator) and *DHS3* “down” were amplified by PCR to direct the integration of exogenous genes into the *DHS3* locus of the *X. dendrorhous* genome. Arrows represent the primers used (Additional file 1: Table S1).

**Figure 4:**
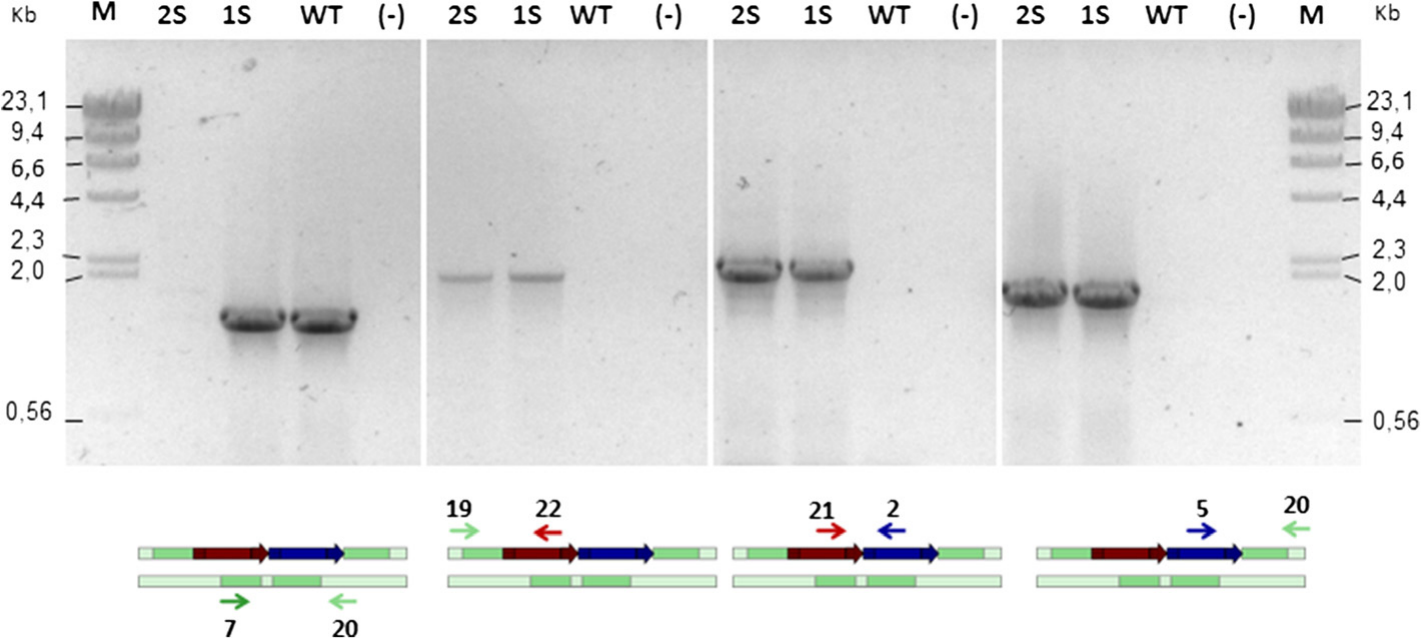
**PCR-based analysis of the *crtS *expression cassette and hygromycin B resistance cassette integration into the genome of *X. dendrorhous*.** PCR analyses of *Xd_*1H1S (1S, one additional *crtS* gene copy), *Xd_*2H2S (2S, two additional *crtS* gene copies), and parental UCD 67–385 (WT) strains, and the negative control without DNA (-). A scheme representing the primers sets that were used (in arrows and numbers according to Additional file 1: Table S1) and the DNA target are under each gel photograph. The scheme shading is in accordance with Figure 3. M: Molecular marker, lambda DNA digested with *Hin*dIII.

Next, we used the above methodology to increase the *crtS* gene dose. A *crtS* expression cassette was constructed by OE-PCR (overlap extension- PCR) and cloned into plasmid pBluescript SK-, resulting in the plasmid pBS-PTEF-*crtS*-Tact. This cassette was integrated in the *DHS3* locus, as its interruption did not cause auxotrophy or greatly affected the carotenogenesis in this yeast. Four DNA fragments (the hygromycin B-resistance selection marker, the *crtS* gene expression cassettes, and the *DHS3* locus “up” and “down” targeting fragments) were individually PCR amplified with primers allowing 50 to 100 bp of overlap between adjacent fragments, and then these fragments were used to co-transform *X. dendrorhous* by electroporation (Figure 3). Four hygromycin B-resistant colonies were obtained. All had the *crtS* gene expression cassettes at the expected locus, as confirmed by PCR analyses with a comprehensive set of primers. One of these four colonies was randomly selected, named strain *Xd_*1H1S (*DHS3*/*dhs3*^::*hph*+*crtS*^, one additional *crtS* gene copy) and subjected to DRM to result in a homozygous strain, *Xd*_2H2S (*dhs3*^::*hph*+*crtS*^/*dhs3*^::*hph*+*crtS*^). The PCR amplification confirmed the insertion of the hygromycin B resistance and *crtS* expression cassettes at the *DHS3* locus in the heterozygous and homozygous transformants are shown in Figure 4.

**Figure 5:**
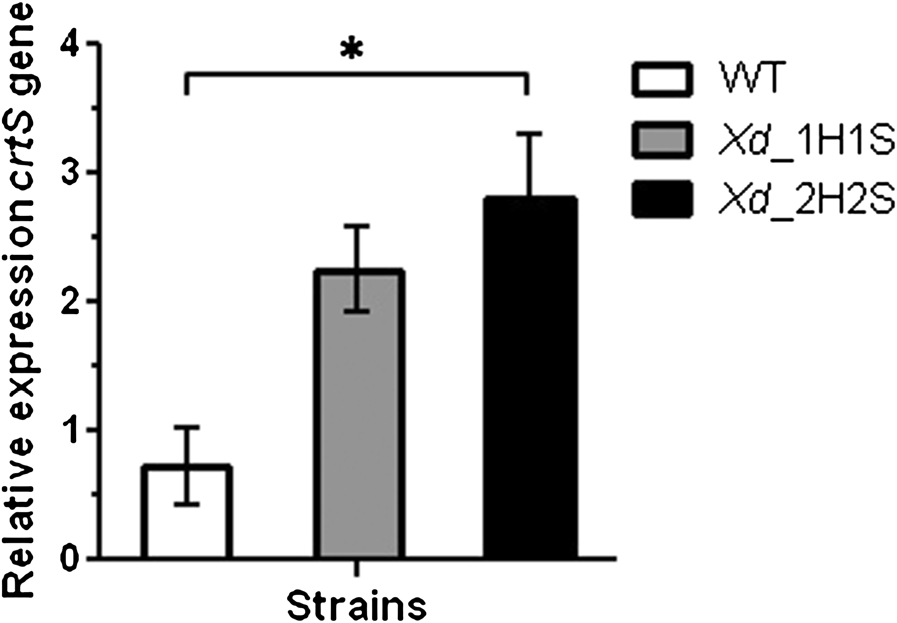
**RT-qPCR expression analysis of *crtS *in the wild-type strain and *crtS-*overexpressing transformants.** The *crtS* transcript level was normalized to the *actin* mRNA and was determined from RNA extracted after 50 h of culture in YM medium from the parental wild-type UCD 67–385 (WT), the heterozygous *Xd_*1H1S (*DHS3*/*dhs3*^::*hph*+*crtS*^) and homozygous *Xd_*2H2S (*dhs3*^::*hph*+*crtS*^/*dhs3*^::*hph*+*crtS*^) strains. Values are the mean ± standard error of at least three independent cultures. (*P ≤ 0.05; Student’s *t* test).

To assess whether the increase in the *crtS* gene dose effectively leads to an increase in the *crtS* mRNA levels, RT-qPCR analysis was performed for the wild-type, heterozygous and homozygous transformants grown under the same conditions. Total RNA was extracted after 50 h of culture (late exponential phase of growth) of each strain as it was observed that the *crtS* mRNA level reaches its maximum level at this stage [32,33]. The relative *crtS* expression was normalized to the expression of the *actin* gene [34], and RT-qPCR analysis revealed that the expression of the *crtS* gene was increased approximately three fold in the *Xd*_2H2S strain with respect to the wild-type strain (UCD-67-385) at this same growth stage (Figure 5).

### Carotenoid production

To the naked eye, different pigmentation phenotypes among the wild-type, *Xd_*1H1S and *Xd*_2H2S strains could be noted. To confirm this observation, the carotenoid content and composition of these strains were analyzed. Total carotenoids were extracted from cell pellets from 24, 50 and 96-h-old yeast cultures (early exponential, late exponential and late stationary phase of growth, respectively) grown at 22°C in YM (yeast malt broth) medium with constant agitation. Carotenoids were extracted, quantified by absorbance at 465 nm and their composition was analyzed by RP-HPLC. The results are summarized in Table 1. Although the total carotenoid production is not affected in strains that overexpress *crtS*, it can be observed that after 50 and 95 h of culture, the percentage of astaxanthin in relation to the other carotenoids is higher in strains expressing additional *crtS* gene copies.

**Table 1:** Carotenoids in wild-type and transformant strains in ppm (μg per g of dry yeast)

	***X. dendrorhous *strain [ppm (%)]**								
	**UCD 67–385**			***Xd_*1H1S**			***Xd_*2H2S**		
	**( *DHS3 */ *DHS3 *)**			**( *DHS3 */*dhs3***^**:: *hph *+*crtS***^**)**			**(*dhs3***^**:: *hph *+*crtS***^***/dhs3***^**:: *hph *+*crtS***^**)**		
**Cultivation time (h)**	**25**	**50**	**96**	**25**	**50**	**96**	**25**	**50**	**96**
**Carotenoid:**									
Total carotenoids	**22.70** **±** **11.16**	**34.31** **±** **15.87**	**189.32** **±** **86.51**	**14.35** **±** **11.71**	**64.21** **±** **1.13**	**188.37** **±** **6.05**	**12.98** **±** **4.93**	**84.79** **±** **28.83**	**197.85** **±** **34.65**
	**(100)**	**(100)**	**(100)**	**(100)**	**(100)**	**(100)**	**(100)**	**(100)**	**(100)**
Beta-carotene	ND	4.37 ± 1.25	6.90 ± 0.57	ND	7.4 ± 0.01	3.93 ± 0.08	ND	2.36 ± 0.29	0.72 ± 0.22
		(13)	(4)		(12)	(2)		(3)	(1)
Equinenone^†^	ND	1.36 ± 0.31	7.01 ± 0.46	ND	2.84 ± 0.01	3.84 ± 0.05	ND	0.54 ± 0.32	0.88 ± 0.03
		(4)	(4)		(4)	(2)		(1)	(1)
OH-equinenone^†^	ND	4.65 ± 0.38	11.47 ± 1.10	ND	5.79 ± 0.01	7.29 ± 0.04	ND	8.85 ± 0.31	1.06 ± 0.16
		(14)	(6)		(9)	(4)		(10)	(1)
Cantaxanthin^†^	ND	0. 46 ± 0.12	2.24 ± 0.90	ND	0.73 ± 0	1.41 ± 0.05	ND	0.39 ± 0.12	0.83 ± 0.21
		(1)	(1)		(1)	(1)		(1)	(1)
Phoenicoxanthin^†^	0.46 ± 0.39	3.99 ± 0.37	28.41 ± 3.53	0.5 ± 0.41	6.65 ± 0.01	15.93 ± 0.01	ND	5.50 ± 0.41	11.08 ± 0.07
	(2)	(12)	(15)	(3)	(10)	(8)		(6)	(6)
Astaxanthin^†^	21.97 ± 0.62	18.81 ± 1.77	128.04 ± 5.06	13.85 ± 0.41	38.05 ± 0.04	154.13 ± 0.09	12.98 ± 0	65.84 ± 0.81	179.21 ± 0.52
	(97)	(55)	(68)	(97)	(59)	(82)	(100)	(78)	(96)
Lycopene	ND	ND	ND	ND	ND	ND	ND	ND	0.51 ± 0.15
									(1)
Neurosporene	ND	0.10 ± 0.04	ND	ND	0.54 ± 0.01	ND	ND	ND	ND
		(0)			(1)				
Torulene^*^	ND	0.07 ± 0.04	ND	ND	0.206 ± 0.01	ND	ND	ND	0.45 ± 0.03
		(0)			(1)				(1)
OH-keto-torulene^†*^	ND	0.47 ± 0.20	0.76 ± 0.52	ND	1.72 ± 0.02	1.85 ± 0.02	ND	0.95 ± 0.24	1.49 ± 0.26
		(1)	(1)		(2)	(1)		(1)	(1)

ND: Not detected. Table values correspond to the average result from three independent cultures ± standard deviations. Percentage relative to total carotenoids is indicated in parentheses. ^†^: hydroxy or/and keto carotenoid, ^*^: monocyclic carotenoid.

## Discussion

There are a limited number of currently available molecular tools to manipulate and transform *X. dendrorhous*. Until this report, transformation of *X. dendrorhous* usually required the construction of a plasmid for transformation [17,25-27], which involves several conventional steps of sequential cloning such as DNA amplification, endonuclease digestion, *in vitro* ligation and transformation. If the number of expression cassettes to incorporate in a host is increased, these processes will be time consuming and will depend on the availability of restriction sites in the cloning vector. In contrast, the DNA assembler method requires only DNA fragments with homologous ends. We find that this methodology works in *X. dendrorhous* as we transformed this yeast with three DNA fragments to integrate a hygromycin B resistance cassette into its genome. We also increased the number of *crtS* gene copies in this yeast by transforming it with four DNA fragments to be assembled (5,098 kb in total) and integrated in the yeast genome. We conclude that this methodology can be employed to engineer *X. dendrorhous* to interrupt or increase gene copy number of several combinations of genes in only one transformation event.

In previous studies attempting to overexpress genes in *X. dendrorhous*, expression cassettes were integrated into ribosomal DNA (rDNA) [17-20]. There are approximately 60 rDNA copies in *X. dendrorhous*[24], which favors multiple integrations of the transformant DNA into the genome. However, due to the tandem repeats in this region, the resulting strains are typically unstable as the integrated genes may be lost by homologous recombination. Additionally, the use of this region makes it difficult to evaluate the effect of gene dose on the production of carotenoids, as it is difficult to quantify the number of integrated cassette copies. For these reasons, we excluded the rDNA region as a target site for integration and focused on integration and resultant target site disruption in a locus that does not strongly modify the yeast physiology. From the analyzed targets, the disruption of locus *DHS3* fulfilled this requirement, so it was chosen to integrate the additional *crtS* gene copies.

In this work, we studied the effect of altering the metabolic flux between beta-carotene and astaxanthin by increasing the *crtS* gene dose. Although the total carotenoid content in strains with one or two additional *crtS* gene copies was not substantially modified (Table 1), an increased proportion of astaxanthin relative to the total carotenoid content was obtained in the strain with two additional *crtS* gene copies compared to the wild-type strain. Astaxanthin represented 96% versus 68% of the carotenoids in these strains after 96 h of culture. In addition, reduced proportions of beta-carotene and intermediate xanthophylls formed during the synthesis of astaxanthin from beta-carotene such as echinenone, hydroxyechinenone, canthaxanthin and phoenicoxanthin. These observations suggest that astaxanthin synthesis is limited by the amount of astaxanthin synthase substrate, beta-carotene. Thus, to achieve a significantly higher level of astaxanthin production, it is also necessary to increase the synthesis of beta-carotene. In agreement with this assumption, it has been observed that the overexpression of the *crtYB* gene in *X. dendrorhous* resulted in increased beta-carotene production [17] and recently, the simultaneous overexpression of the *crtYB* and *crtS* genes in a *X. dendrorhous* strain created via random mutagenesis that already overproduced astaxanthin resulted in a higher astaxanthin content compared to the parental strain (5,300 ppm) [20]. The methodology described in this work should be a helpful tool to evaluate the consequences of overexpressing different combinations of genes involved in carotenoid production in *X. dendrorhous*.

Although beta-carotene was almost completely exhausted in the transformant strain that overexpressed *crtS*, phoenicoxanthin, the xanthophyll precursor to astaxanthin, was still present, although its proportion was reduced from 15% to 6% after 96 h of culture in the wild-type and in the *Xd_*2H2S strains, respectively. As astaxanthin synthase is a cytochrome P450 enzyme, it may also be necessary to increase the cytochrome P450 reductase [25] gene dose to enhance the activity of this enzyme.

The DNA assembler methodology that has been effective and successful in *X. dendrorhous* in this study should be useful to overexpress several genes simultaneously to favor the synthesis of carotenoid precursors such as geranylgeranyl pyrophosphate or genes involved in the mevalonate pathway [35] in combination with carotenogenic genes. Thus, using different promoters in the construction of the gene expression cassettes, it should be possible to increase and modulate the production of carotenoids in *X. dendrorhous*.

## Conclusions

The DNA assembler method is a successful technique to transform *X. dendrorhous*. This technique allowed an increase in the *crtS* gene copy number in the *X. dendrorhous* genome. The overexpression of this gene did not significantly change the total carotenoid production, but there was an increase in the astaxanthin fraction of carotenoids.

As DNA assembler requires only DNA fragments with homologous ends, this technique could be useful to quickly and easily overexpress several genes simultaneously in *X. dendrorhous*, saving numerous steps involved in conventional cloning methods.

## Methods

The experiments performed in this work were approved by the Facultad de Ciencias – Universidad de Chile, Ethics Committee.

### Microorganisms, plasmids, media, and enzymes

The strains and plasmids that were used and built in this work are listed in Table 2. The wild type *X. dendrorhous* strain UCD 67–385 (ATCC 24230) was used as a parental strain for transformation experiments and *E. coli* DH-5α was used as a host for plasmid propagation.

**Table 2:** Strains and plasmids used and built in this work

	**Genotype or relevant features**	**Source or reference**
Strains:		
*E. coli*:		
DH-5α	F- φ80d lacZΔM15Δ (lacZY-argF) U169 deoR recA1 endA1 hsdR17(rk- mk+) phoA supE44l- thi-1 gyrA96 relA1	[37]
*X. dendrorhous*:		
UCD 67-385	ATCC 24230, wild-type. Diploid strain [31].	ATCC
*Xd_*1H	(*DHS3*/*dhs3*^::*hph*^). Heterozygote transformant derived from UCD 67–385 containing an allele of the *DHS3 locus* with a deletion and a hygromycin B resistance cassette.	This work
*Xd_*2H	(*dhs3*^::*hph*^/*dhs3*^::*hph*^). Homozygote transformant derived from *Xd_*1H by DRM [30] with a deletion and a hygromycin B resistance cassette in both alleles of the *DHS3 locus*.	This work
*Xd_*1H1S	(*DHS3*/*dhs3*^::*hph*+*crtS*^). Heterozygote transformant derived from UCD 67–385 with a deletion in one *DHS3* allele and bearing the hygromycin B resistance cassette and the *crtS* gene expression cassette.	This work
*Xd_*2H2S	(*dhs3*^::*hph*+*crtS*^/*dhs3*^::*hph*+*crtS*^). Homozygote transformant derived from *Xd_*1H1S by DRM with a deletion in both *DHS3* alleles and bearing the hygromycin B resistance cassette and the *crtS* gene expression cassette.	This work
Plasmids:		
pBluescript SK- (pBS)	ColE1 ori; AmpR; cloning vector for blue-white selection	Stratagene
pMN-*hph*	pBS containing a cassette of 1.8 kb bearing the resistance Hygromycin B (*hph*) gene under EF-1 α promoter and *gpd* transcription terminator of *X. dendrorhous* in its *Eco*RV site.	[30]
p*Xd_*Ex_*crtS*	pBS containing the cDNA encoding the *X. dendrorhous crtS* gene in its *Eco*RV site.	[26]
pBS-PTEF-*crtS*-Tact	pBS containing a 2,367 bp cassette bearing the cDNA encoding the *X. dendrorhous crtS* gene under EF-1α promoter and the *actin* transcription terminator from *X. dendrorhous* in the *Eco*RV site.	This work

ATCC: American Type Culture Collection.

*DHS3* [GenBank: JN849376.1]: locus downstream of the *X. dendrorhous HIS3* gene [GenBank: JN849374.1].

*X. dendrorhous* strains were grown at 22°C with constant agitation in YM medium (1% glucose, 0.3% yeast extract, 0.3% malt extract and 0.5% peptone) or minimal MM_V_ + 2% glucose medium [36]. Yeast transformant selection was performed on YM 1.5% agar plates supplemented with 15 μg/ml hygromycin B. *E. coli* strains were grown with constant agitation at 37°C in Luria-Bertani (LB) medium and supplemented with 100 μg/ml ampicillin for plasmid selection and 80 μg/ml X-gal (5-bromo-4-chloro-3-indolyl-β-D-galactopyranoside) for recombinant clone selection [37].

Enzymes were purchased from Promega (*Taq* DNA pol, restriction enzymes, M-MLV reverse transcriptase) and *Pfu* DNA pol was purchased from Agilent Technologies.

### DNA amplification

All of the nucleotides used in this work were purchased from Alpha DNA (Montreal, Canada) or from Integrated DNA Technologies and are listed in Additional file 1: Table S1. PCR reactions were performed in a final volume of 25 μl containing 2 U of *Taq* DNA pol, 2.5 μl of 10× *Taq* buffer, 0.5 μl of 10 mM dNTPs, 1 μl of 50 mM MgCl_2_, 1 μl of 25 μM of each primer and 10–20 ng of template DNA. Amplification was performed in a DNA thermal cycler 2720 (Applied Biosystems) as follows: initial denaturation at 95°C for 3 min; 35 cycles of denaturation at 94°C for 30 s, annealing at 55°C for 30 s, synthesis at 72°C for 3 min and a final extension step at 72°C for 10 min. Samples were kept at 4°C until use. The amplicons were separated by 0.8% agarose gel electrophoresis in TAE buffer containing 0.5 μg/ml ethidium bromide [37] followed by DNA purification using the Glassmilk method [38] for sequencing or gene expression cassette construction.

To reduce the error rate in DNA amplification for Overlap Extension-PCR (OE-PCR, [39] and DNA fragment amplification for *X. dendrorhous* transformation, *Pfu* DNA pol was used instead of *Taq* DNA pol following the manufacturer’s instructions.

### Cloning and construction of the *crtS* gene expression cassette

The *crtS* gene expression cassette was constructed by Overlap Extension-PCR (OE-PCR) [39] containing 1,674 bp of the cDNA of the *crtS* gene [GenBank: DQ002007.1] under the control of 393 bp of the *X. dendrorhous* EF-1α promoter [26] and 300 bp of the *actin* transcription terminator of [GenBank: X89898.1]. The EF-1α promoter region was amplified by PCR with primers 1 and 2 (Additional file 1: Table S1) using pMN-*hph* as a template (Table 2), and the *actin* gene terminator was amplified from UCD 67–385 genomic DNA using primers 5 and 6 (Additional file 1: Table S1). The *crtS* cDNA was amplified from p*Xd_*Ex_*crtS* (Table 2) with primers 3 and 4 (Additional file 1: Table S1). First, the promoter region was joined to the *crtS* cDNA and then the hybrid product was joined to the *actin* gene transcription terminator by OE-PCR. The resulting cassette was cloned into the *Eco*RV site of plasmid pBS (pBS-PTEF-*crtS*-Tact, Table 2) and was sequenced on both strands using a DYEnamic ET Terminator Kit (Amersham Bioscience) in an ABI 3100 Avant genetic analyzer. DNA sequences were analyzed with Vector NTI Suite 10 (Informax) and bioinformatics programs available online.

### *X. dendrorhous* transformation

*X. dendrorhous* transformation was performed by electroporation according to [40] and [41]. Electrocompetent cells were prepared from an exponential culture with DO_600nm_ = 1.2, cultured in YM medium and electroporated using a BioRad Gene Pulser Xcell with PC and CE modules under the following conditions: 125 mF, 600 Ω, 0.45 kV. The yeast were transformed with 4 μl of a mixture of purified DNA fragments (1 μg of each fragment) that were amplified by PCR with *Pfu* DNA pol and primers with 5′ complementary ends to allow their assembly by homologous recombination. In each transformation event, in addition to the selection marker and/or the *crtS* gene expression cassette, “up” and “down” DNA fragments were included targeting the insertion into the integration site. The hygromycin B resistance and *crtS* gene expression cassettes were amplified from plasmids pMN-Hyg and pBS-PTEF-crtS-Tact, respectively, and the “up” and “down” DNA fragments were amplified from *X. dendrorhous* wild-type genomic DNA. Transformant selection was performed on YM 1.5% agar plates supplemented with 15 μg/ml hygromycin B. As strain UCD 67–385 is diploid [31], heterozygous transformants were obtained. To obtain homozygous transformants, the double recombinant method (DRM) [30] was applied. The transformant strains were confirmed as *X. dendrorhous* by examining the ITS1, 5.8 rRNA gene and ITS2 DNA sequences [42,43].

### RNA extraction, single strand DNA synthesis and RT-qPCR

To measure the relative *crtS* gene expression, total RNA was extracted from 50 h yeast cultures (late exponential phase of growth) grown at 22°C with constant agitation in YM medium. The cell pellets from 5 ml culture aliquots were frozen with liquid nitrogen and stored at -80°C until use. Total RNA extraction from the cell pellets was performed by mechanical rupture with 0.5 mm glass beads (BioSpec) during 10 min of vortexing, followed by the addition of Tri-Reagent (Ambion). The lysate was incubated for 5 min at room temperature and 200 μl of chloroform per ml of Tri-Reagent used was added, mixed, and centrifuged for 5 min at 4,000 × g. Following this centrifugation, the aqueous phase was recovered. The RNA was precipitated by adding two volumes of isopropanol and incubating at room temperature for 10 min. The RNA was washed with 75% ethanol, suspended in RNase-free H_2_O and quantified by absorbance determination at 260 nm in a double beam Shimadzu UV-150-20 spectrophotometer.

The synthesis of cDNA was performed according to the M-MLV reverse transcriptase (Invitrogen) manufacturer’s protocol with 5 μg of total RNA in a final volume of 11 μl. The determination of the relative *crtS* gene expression levels was performed in an Mx3000P quantitative PCR system (Stratagene) using 1 μl of the reverse transcription reaction, 0.25 μM of each primer (Additional file 1: Table S1) and 10 μl of the SensiMix SYBR Green I (Quantace) kit reagent in a final volume of 20 μl. The obtained Ct values were normalized to the respective value of the *actin* gene [GenBank: X89898.1] [44] and expressed using the 2^-ΔC^_T_ method [45,46].

### Carotenoid extraction and RP-HPLC

Carotenoids were extracted from cell pellets from 24, 50 and 96-hours-old yeast cultures (early exponential, late exponential and late stationary phase of growth, respectively) grown at 22°C with constant agitation in YM medium using the acetone extraction method [12]. Total carotenoids were quantified by absorbance at 465 nm using an absorption coefficient of A_1%_ = 2,100. The analyses were performed at least in triplicate, and pigments were normalized relative to the dry weight of the yeast. Carotenoids were separated by RP-HPLC using a reverse phase RP-18 LiChroCART 125–4 (Merck) column with acetonitrile:methanol:isopropanol (85:10:5 v/v) as the mobile phase under isocratic conditions with a 1 ml/min flux. The elusion spectra were recovered using a diode array detector, and carotenoids were identified by their spectra and retention time according to standards.

## Competing interests

The authors declare that they no competing interests.

## Authors’ contributions

GC constructed the *crtS* expression module, performed the *X. dendrorhous* transformations and transformant analyses, carotenoid analyses and gene expression analyses. SB contributed to the RT-qPCR gene expression analyses. MCR and MB contributed to the study design and results analyses. VC participated in the experimental design and coordination. JA conceived the study and participated in its design and coordination. JA, MB, VC drafted the manuscript. All authors read and approved the final manuscript.

## Supplementary Material

Additional file 1: Table S1 Primers used in this work. Click here for file
