# Supplementary material for: Increase in the astaxanthin synthase gene (crtS) dose by in vivo DNA fragment assembly in Xanthophyllomyces dendrorhous
Source: BMC Biotechnol. 2013 Oct 9;13:84. doi: 10.1186/1472-6750-13-84 (PMC3852557; doi:10.1186/1472-6750-13-84)
Supplement: Additional file 1: Table S1 — Primers used in this work. [file 1472-6750-13-84-S1.docx]

## Table S1 - Primers used in this work.

| Nº | Primer | Sequence 5’ to 3’ | Target |
| --- | --- | --- | --- |
| Construction of *crtS* expression cassette: | | | |
| 1 | TEF. 1F | GGCTCATCAGCCGACAGTTC | EF-1α promoter |
| 2 | 21TEF-13crtS. R | **CCAAGATGAACAT**TGAAGCTGTTCGAGATAGATT | EF-1α promoter |
| 3 | 13TEF-21crtS. F | **TCGAACAGCTTCA**ATGTTCATCTTGGTCTTGCTC | *crtS* cDNA |
| 4 | 21 crtS- 13ACTt. R | **AGACTTTGTTGAC**TCATTCGACCGGCTTGACCTG | *crtS* cDNA |
| 5 | 21ACTt-13crtS. F | **GCCGGTCGAATGA**GTCAACAAAGTCTTTCTATCC | *actin* terminator |
| 6 | 21 actT. R | TATTTATTACGTCTAATGTA | *actin* terminator |
|  |  |  |  |
| *X. dendrorhous* transformation: | | | |
| 7 | x.His.up.F | TTGCAACCTCGAGTTGATAG | *DHS3* up region |
| 8 | x.His.up.R | TTGGTCTCGTCTTTGTTCGC | *DHS3* up region |
| 9 | x.H.up-50TEF.R | **GACGATCTAGGCAAAGAGCTTGTGTCGGATGAACTGTCGGCTGATGAGCC**TTGGTCTCGT | *DHS3* up region with 3’ homologous end with 5’ EF-1α promoter. |
| 10 | TEF-50.x.H.up.F | **TCGTCTTCTTTTTCGATCTAAGGCACAGACGCGAACAAAGACGAGACCAA**GGCTCATCAG | EF-1α promoter with 3’ homologous end with 5’ *DHS3* up region. |
| 11 | gpd-50xH.dw. R | **ATATCTGAAGGGAAAAAAGAAAAACGAAAGAACCTCACTCAACCGATGTC**ATCATGAGAG | *gpd* terminator with 3’ homologous end with 5’ *DHS3* down region. |
| 12 | xH.dw-50gpdT. F | **TTCAATCACATCTGTTGACCATCACCATCATCTCCGTCATCTCTCATGAT**GACATCGGTT | *DHS3* down region with 3’ homologous end with 5’ *gpd* terminator. |
| 13 | xHis.dw. F | GACATCGGTTGAGTGAGGTT | *DHS3* down region |
| 14 | xHis.dw. R | AATTGTGATGTGTGACGGGC | *DHS3* down region |
| 15 | 10TEF-50gpd.F | **TTCAATCACATCTGTTGACCATCACCATCATCTCCGTCATCTCTCATGAT**GGCTCATCAG | EF-1α promoter with 3’ homologous end with 5’ *gpd* terminator. |
| 16 | ACTt-50xHis.R | **ATATCTGAAGGGAAAAAAGAAAAACGAAAGAACCTCACTCAACCGATGTC**TATTTATTAC | *actin* terminator with 3’ homologous end with 5’ *DHS3* down region |
| 17 | xHis.49actT.F | **ATGAGATTTATTCAACTACTAAATAACGATACATTAGACGTAATAAATAG**ACATCGGTTG | *DHS3* down region with 3’ homologous end with 5’ *actin* terminator |
| 18 | gpd.R | ATCATGAGAGATGACGGAGA | *gpd* terminator |
|  |  |  |  |
| *X. dendrorhous* transformant analysis: | | | |
| 19 | 1.out.xHis.F | GAGAGGAAGATAGAGGAGAG | *DHS3 locus* |
| 20 | 2.out.xHis. R | CGTTCAGGAGTGTGAAAACG | *DHS3 locus* |
| 21 | HF | ATGAAAAAGCCTGAACTCACC | *hph* gene |
| 22 | HR | CTATTCCTTTGCCCTCGGAC | *hph* gene |
| 23 | 1.in.xHis.F | AGATCATTAGCAGGTGAGCC | *DHS3 locus* |
| 24 | 2.in.xHis.R | GTTTACCACCTGCCTCAAAC | *DHS3 locus* |
| 25 | crtSmd.F | CCTTTGACTCAAGGATTAGC | *crtS* gene |
| 26 | crtSmd.R | GCTAATCCTTGAGTCAAAGG | *crtS* gene |
|  |  |  |  |
| RT-qPCR, *crtS* gene expression (The pairs of primers used had efficiency greater than 95% as determined by standard curves with a correlation coefficient of R^2^ ≥ 0.996): | | | |
| 27 | crtS.RT.3.1.F | CTTGAGGACGTAACAGACTC | *crtS* gene |
| 28 | crtS.RT.3.2. R | ACAAGAAGGCCTTCATCTCG | *crtS* gene |
| 29 | mActF-RT | CCGCCCTCGTGATTGATAAC | *actin* gene |
| 30 | mActR-RT | TCACCAACGTAGGAGTCCTT | *actin* gene |

F and R in the primer name indicate the primer orientation. The overlapping nucleotides in primers are in bold.
